# The Terrestrial Silica Pump

**DOI:** 10.1371/journal.pone.0052932

**Published:** 2012-12-31

**Authors:** Joanna C. Carey, Robinson W. Fulweiler

**Affiliations:** 1 Department of Earth and Environment, Boston University, Boston, Massachusetts, United States of America; 2 Department of Biology, Boston University, Boston, Massachusetts, United States of America; University of Delhi, India

## Abstract

Silicon (Si) cycling controls atmospheric CO_2_ concentrations and thus, the global climate, through three well-recognized means: chemical weathering of mineral silicates, occlusion of carbon (C) to soil phytoliths, and the oceanic biological Si pump. In the latter, oceanic diatoms directly sequester 25.8 Gton C yr^−1^, accounting for 43% of the total oceanic net primary production (NPP). However, another important link between C and Si cycling remains largely ignored, specifically the role of Si in terrestrial NPP. Here we show that 55% of terrestrial NPP (33 Gton C yr^−1^) is due to active Si-accumulating vegetation, on par with the amount of C sequestered annually via marine diatoms. Our results suggest that similar to oceanic diatoms, the biological Si cycle of land plants also controls atmospheric CO_2_ levels. In addition, we provide the first estimates of Si fixed in terrestrial vegetation by major global biome type, highlighting the ecosystems of most dynamic Si fixation. Projected global land use change will convert forests to agricultural lands, increasing the fixation of Si by land plants, and the magnitude of the terrestrial Si pump.

## Introduction

Global silicon (Si) cycling regulates atmospheric carbon dioxide (CO_2_) concentrations via several well-known mechanisms, particularly chemical weathering of mineral silicates [1], occlusion of carbon (C) to soil phytoliths [2], and the oceanic biological Si pump [3]. The vast majority of research on Si cycling has focused on the oceans, where Si-replete diatoms sequester 240 Tmol Si yr^−1^ [4]. Diatoms are also a critical component of the global C cycle, accounting for 35–75% of marine net primary production (NPP) [4] and serving as efficient exporters of C to the benthos [5].

However, similar to diatoms, terrestrial vegetation can also sequester large amounts of Si. Si is considered a ‘quasi-essential’ nutrient for plants [6]. While most plants can grow in Si-deplete soils, plant fitness is markedly improved with Si amendments [7]. In fact, Si is the only element that has been shown to never be toxic to plants even in high doses [7]. Silicon protects plants from a variety of abiotic and biotic stresses, including desiccation, predation, fungal attack, and heavy metal toxicity [6]–[8]. Ultimately, Si plays a critical role in plant defense [6], which we argue better facilitates plants to perform essential services, particularly C sequestration.

Si concentrations in land plants range over two orders of magnitude (<0.1 to over 10% by dry weight (by wt.)), the largest range of any element [6]. All photosynthetic plants contain some Si within their tissue, often in concentrations equal to or greater than other macronutrients, such as nitrogen (N), phosphorus (P), and potassium (K) [8]. Plants are typically broken into three modes of Si uptake: rejective, passive accumulators, or active accumulators [7]. Plants whose Si uptake is greater than that which would passively be taken up through the transpiration stream are defined as active accumulators and typically contain >0.46% Si (or 1% SiO_2_) by wt. [7], [9], [10].

Plants take up dissolved silica (DSi) (H_4_SiO_4_) from soil solution via their roots. DSi is transported via the xylem for eventual deposition in transpiration termini. Upon incorporation into organisms, biogenic silica (BSi) is formed, creating siliceous bodies in cell walls known as phytoliths. While the ultimate source of Si in the biosphere is chemical weathering of mineral silicates, BSi is 7 to 20 times more soluble than mineral silicates [11], resulting in BSi being an important source of DSi on biological time scales [12]. The dynamic cycling of Si on its path from land to sea has only recently been documented [11], [13], demonstrating that the biological component of the global Si cycle is driven not only by diatoms, but also by terrestrial organisms [14].

To date, the amount of Si fixed by land plants has only been estimated at the global scale [13], [15], never by major terrestrial ecosystem type. Because certain plants, such as grasses, fix more Si than others, fixation of Si on land is far from uniform. Thus, we distinguish the hot spots of Si fixation, by calculating the amount of Si fixed by biome type (Table 1). Using this information, we calculate the percentage of terrestrial NPP done via active-Si accumulating organisms. To do this, we categorized Si shoot/leaf concentrations of 32 orders of angiosperms, gymnosperms, and mosses into 11 biome categories. Taxonomic order was chosen to categorize plants, as it accounts for the majority (67%) of the variability of Si concentrations within plant tissue [16].

**Table 1:** Amount of Si fixed by major biome type. Bold indicates on average, active Si accumulation, indicated by concentrations >0.46% Si by wt. [7].

Biome Type	NPP (C)×10^3^(Tmol yr^−1^)		Avg %Siby wt.	Si:C	Si(Tmol yr^−1^)
Tropical wet andmoist forest	0.69	0.30		0.006	4.48
Tropical dry forest	0.40	0.28		0.006	2.35
Temperate forest	0.50	0.21		0.004	2.22
Boreal forest	0.53	0.23		0.005	2.65
Tropical woodlandand savanna	0.93	**1.13**		0.024	22.19
Temperate steppe	0.41	**1.53**		0.032	13.26
Desert	0.12	0.26		0.006	0.65
Tundra	0.12	**1.07**		0.023	2.66
Wetland	0.32	**0.62**		0.013	4.16
Cultivated land	1.01	**1.37**		0.029	29.41
Rock and ice	0	0.00		0.000	0
*Average*		0.64		0.014	
*Total*	5.02				**84.03**
*Std Dev*		0.32		0.006	28.86

Si concentrations from Hodson et al. [16]. Biome categories from Houghton and Skole [17].

## Materials and Methods

Leaf tissue Si concentrations were taken from supplemental data assembled in the meta-analysis completed by Hodson et al. (2005). From this dataset, 32 orders of angiosperms, gymnosperms, and mosses (representing 528 species) were assigned to a specific biome type. Fifteen orders fell into two different biome types (e.g. Pinales – temperate and boreal forest biomes). The order Poales (i.e. grasses, sedges, and rice) was the only order that fell into more than two biome types.

Given that average C concentrations in biomass consistently range from 45 to 50% [17], we assumed a wood C concentration of 47%. Dividing the calculated biome-specific average Si concentrations by 47% allowed us to calculate Si:C ratios for each biome (Table 1). In turn, our biome-specific Si:C ratios were multiplied by the known amount of C in each biome [17], creating a constrained estimate of the Si fixed by ecosystem type (Table 1).

Error estimates for the four forest biomes and the wetland biome were calculated by taking the standard deviation of the Si concentrations in the leaf/shoot tissue. Average Si concentration of the ‘tropical woodland and savanna’ biome was weighted for vegetation type (grasses accounted for 70%, herbaceous shrubs and trees accounted for 30% [18]). Error estimates associated with this biome include a sensitivity analysis of ±20% of each plant type. Likewise, average Si concentration of cultivated crops was weighted to account for the mass of the top four crops produced in 2010 [19] (Table 2). Average Si concentration of the tundra biome used an non-weighted average of the Poales (sedges), Sphagnales (mosses), and Ericales (herbaceous shrubs) orders. Average Si concentration of the temperate steppe biome included only Poales (grasses) as the predominant vegetation type.

**Table 2:** Average Si concentration of cultivated crop biome, weighted by the mass of the four most produced crops [19].

Order	Genus	% Siby wt	Mass of Crop(Million Tons)	Si(Million tons)
Poales	Saccharum	0.54	1685	910
Poales	Triticum	2.42	650	1573
Poales	Zea	0.79	844	667
Poales	Oryza	3.17	672	2130
*Total*			3851	5280
*Average Si concentration*				**1.37**

Si fixed through land use change via increased agriculture was calculated by creating a correction factor that accounted for changes in land area of biome categories [20]. Correction factors were multiplied by the amount of Si currently fixed in each biome category in order to estimate the amount of Si fixed by 2050.

## Results

The amount of Si fixed annually by the 11 major biome types found on Earth was calculated (Table 1). Unsurprisingly, the least amount of Si was found in the rock and ice, and desert biomes (<1 Tmol yr^−1^). Cultivated lands contain more Si than any other biome (29 Tmol yr^−1^), due to both high average Si content of the most common cultivated crops (Table 2) and the large amount of biomass present on cultivated lands (Tables 1, 2).

While Si typically is deposited in the foliage, or leaves of trees, wood can also contain appreciable amounts of Si. Tropical forests contain relatively high levels of Si within their wood (Avg. 0.31% by wt.) [21], [22], roughly four times more than the wood of temperate and boreal forests (Avg. 0.08% by wt.) [23]. In order to account for the different allocation of Si within various components of forest ecosystems, forest biome average Si concentrations were weighted to account for 30% of forest NPP being attributed to foliage, with the remainder allocated to woody biomass [24].

**Figure 1:**
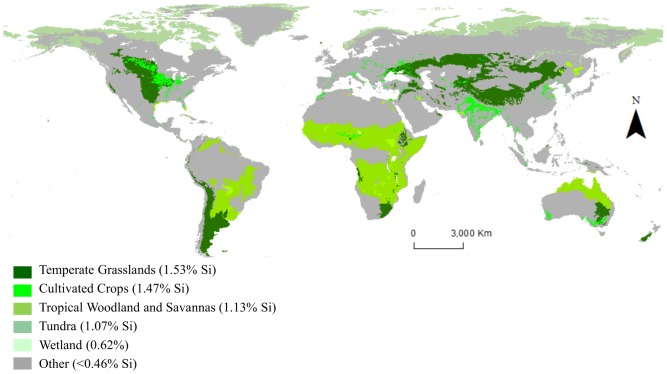
Global land cover by Si accumulating crops. Shades of green are directly proportional to degree of active Si accumulation (i.e., darker green indicates more Si accumulation). Gray area indicates land cover by biomes of non-active Si accumulators (<0.46% Si by dry wt.) (Table 1). Data from combination of FAO GeoNetwork Global Land Cover Distribution 2007 raster data layer with a resolution of 5-arc minutes, and ESRI, which modified the layer originally created by Olson et al. (2001). Map created using ArcMap 10.0 from ESRI.

Active Si accumulation, indicated by average Si concentrations >0.46% by wt., was observed in five biomes: tropical woodland and savanna, temperate steppe, tundra, wetland, and cultivated lands (Figure 1). The elevated concentrations in these biomes were driven mostly by the predominance of the order Poales (e.g. grasses, sedges, and rice). These five biomes, which exhibit active Si accumulation, account for 55% of terrestrial NPP (Table 1). Thus, we conclude that just over half of terrestrial NPP is done by active-Si accumulating organisms, rivaling that of oceanic phytoplankton [17]. Combining our results with the estimate that 43% of ocean NPP is via Si-replete diatoms [4], we find that 59 Gton C yr^−1^, or 49%, of current global NPP is conducted by active Si-accumulating organisms.

In order to provide a check of our results, we compared our total amount of Si fixed by land plants with those from previous studies. Summing our values of Si fixed by biome type, we find that 55–113 Tmol Si yr^−1^ is fixed globally in the terrestrial biosphere (Avg. 84±29 Tmol Si yr^−1^). Our value is within the bounds of that provided by Conley (2002) (60–200 Tmol yr^−1^) and almost identical to the global estimate provide by Laruelle et al. (1999) (80 Tmol yr^−1^), providing confidence in our estimates.

## Discussion

Human activities have already altered the amount of Si fixed in aquatic ecosystems. At the same time that the fluxes of N and P to coastal systems have markedly increased [25], river damming has led to significant declines in the amount of Si exported to coastal waters [26]. Combined, these two factors have increased the concentrations of N and P relative to Si, altering the ratio of N:P:Si in marine waters and inducing Si-limitation in several systems [27]–[29]. Such Si-limiting conditions can shift the phytoplankton species composition from diatom to non-diatom species, altering the base of the marine tropic structure [30], [31]. Importantly, because diatoms are more efficient C exporters compared to other types of phytoplankton [5], shifting phytoplankton community structure away from diatoms reduces the amount of C exported to the benthos via the ocean biological pump.

Similarly, we hypothesize that land use change will also alter the magnitude of Si fixed in the terrestrial biosphere, although in the opposite direction. Because both cultivated crops and pasture land (included in grasslands) are the biomes of most Si sequestration, increases in agricultural production will increase the amount of BSi fixed by land plants. Our analysis shows that cultivated crop lands currently account for 35% of the BSi fixed on land each year. Area of crop and pasture land is projected to increase by 8.9×10^6^ km^2^, or 18%, by 2050 [20]. Assuming conversion of tropical forests to agricultural land, we calculate that an additional 7.5 Tmol Si yr^−1^ will be fixed by 2050, an increase of 9% from current levels. Thus, increased food production to feed a larger global population will heighten the magnitude of the terrestrial Si pump, as more BSi will be produced on land.

Because the Si fixed via crop cultivation is transported around the globe in large quantities, agriculture has recently been highlighted as an overlooked component of the global biological Si cycle [32]. Ultimately, much of the BSi stored in crops will be transferred to human population centers, or urban areas, which have been shown to export more Si than their forested counterparts [33]. The heightened production of the more-soluble biogenic Si within the biosphere may increase the capacity of urban systems to serve as sources of Si to coastal waters [34]. In turn, it is possible that Si-limiting conditions downstream of urban centers are buffered by the terrestrial Si pump and not as high as they would be in its absence. We hypothesize that additional Si fluxes from urban centers have the potential to improve the balance to N:P:Si ratios, at least in part, mitigating Si-limitation of nutrient-rich receiving waters.

However, the fate of the cultivated and harvest BSi in crops deserves further research attention, as environmental management practices, such as wastewater treatment, sludge disposal, and farming techniques will ultimately dictate the fate of the additional BSi produced by land plants [32]. In addition, the storage of sediment BSi and subsequent release as DSi are not directly connected processes, as they can occur over variable periods of time [35]. Soil pools contain at least two orders of magnitude more BSi than vegetation pools [13], highlighting that much of the BSi produced on land will be stored in the soils, rather than exported directly via rivers. While agricultural production will increase the amount of Si fixed by land plants, centuries of continual crop harvests have been shown to deplete soil pools of BSi [32], [35], [36]. Thus, conversion of more land for agricultural production could deplete soil BSi reserves over time, which could ultimately impact the magnitude of the terrestrial Si pump and long-term fluxes of Si to coastal systems.
